# Investigation of a Potential Zoonotic Transmission of Orthoreovirus Associated with Acute Influenza-Like Illness in an Adult Patient

**DOI:** 10.1371/journal.pone.0025434

**Published:** 2011-10-13

**Authors:** Kaw Bing Chua, Kenny Voon, Meng Yu, Canady Keniscope, Kasri Abdul Rasid, Lin-Fa Wang

**Affiliations:** 1 National Public Health Laboratory, Sg. Buloh, Selangor, Malaysia; 2 International Medical University, Bukit Jalil, Kuala Lumpur, Malaysia; 3 Australian Animal Health Laboratory, Commonwealth Science and Industry Research Organisation Livestock Industries, Geelong, Victoria, Australia; Veterinary Laboratories Agency, United Kingdom

## Abstract

Bats are increasingly being recognized as important reservoir hosts for a large number of viruses, some of them can be highly virulent when they infect human and livestock animals. Among the new bat zoonotic viruses discovered in recent years, several reoviruses (***r***espiratory ***e***nteric ***o***rphan viruses) were found to be able to cause acute respiratory infections in humans, which included Melaka and Kampar viruses discovered in Malaysia, all of them belong to the genus *Orthoreovirus*, family *Reoviridae*. In this report, we describe the isolation of a highly related virus from an adult patient who suffered acute respiratory illness in Malaysia. Although there was no direct evidence of bat origin, epidemiological study indicated the potential exposure of the patient to bats before the onset of disease. The current study further demonstrates that spillover events of different strains of related orthoreoviruses from bats to humans are occurring on a regular basis, which calls for more intensive and systematic surveillances to fully assess the true public health impact of these newly discovered bat-borne zoonotic reoviruses.

## Introduction

Up-to-date, approximately 1400 pathogen species are recognized to infect humans. Although fewer than 200 of these are viruses, on average, more than two new species of viruses infecting humans are reported worldwide every year and novel viruses, especially RNA viruses, are more likely to cause future emerging infections [1]. Emerging novel viruses pose a major public health concern, whether causing massive progressive pandemic disease such as human immunodeficiency viruses or more transient events such as the Nipah virus in 1998/1999 and SARS coronavirus in 2002/2003 [2], [3], [4].

Acute respiratory infections constitute the most common and widespread human infectious diseases, especially amongst patients seen at the outpatient clinics. Despite the application of various diagnostic tests, the aetiological agents of a substantial proportion of acute respiratory illnesses are still not known. Novel viruses with the ability to cause respiratory infections and spread via the respiratory route, such as the SARS coronavirus and pandemic influenza A (H1N1), are of particular public health interest because of their pandemic potential and their ability of rapid spread via aerosol or droplet. The recent isolations of several novel zoonotic orthoreoviruses from humans with acute respiratory illness and the demonstration of their human-to-human transmission highlights the potential of emergence of a pandemic strain via mutation and/or re-assortment of their segmented double-stranded RNA genomes [5], [6], [7].

Viruses belonging to the family *Reoviridae* are a large and diverse group of non-enveloped viruses with segmented double-stranded RNA genomes [8]. The viruses within this family are taxonomically classified into 12 genera in part depending on the number of their dsRNA genomes. Members of the genus *Orthoreovirus* contain 10 genome segments and have been isolated from a broad range of mammals, birds and reptiles. Viruses under this genus are divided into two phenotypic groups, fusogenic and non-fusogenic, based on the ability of the virus to cause cell-cell fusion and formation of giant syncytial cells [9], [10]. The mammalian orthoreoviruses (MRV) are non-fusogenic whereas the remaining members of the genus are fusogenic, including avian orthoreoviruses (ARV), baboon orthoreoviruses (BRV), reptilian orhtoreoviruses (RRV) and orthoreoviruses isolated from bats [11], [12]. MRV infection in humans has been shown to be fairly common and the infections are often shown to be asymptomatic or associated with mild, self-limiting respiratory or gastrointestinal illness in infants and children [9]. However, infections associated with fusogenic orthoreoviruses have been shown to be of serious consequences ranging from neurological illness in baboon and snakes to pneumonia and death in chickens [9].

Since 2006, two fusogenic orthoreoviruses, Melaka virus and Kampar virus, have been independently isolated in Malaysia from respiratory samples of patients with acute respiratory illness though no death has been reported [5], [6]. A similar virus was discovered in Hong Kong from a patient who visited Bali a few days before the onset of the severe respiratory illness [7]. All of these newly isolated orthoreoviruses are genetically closely related to the Nelson Bay virus and Pulau virus, both of bat origin [11], [13]. Due to the high genetic similarity of genome sequences and strong cross reactivity at the serological level [6], it has been proposed, after recent consultation with the International Committee on Virus Taxonomy, that all of these viruses be placed under a new species, *Pteropine orthoreovirus* (PRV), replacing the existing species, *Nelson Bay orthoreovirus*. The new name has a better fit with other existing species names in the genus such as *Avian orthoreovirus* (ARV) and *Mammalian orthoreovirus* (MRV).

Epidemiological investigation by screening of sera collected from human volunteers on the Pulau Island in Malaysia revealed that 14 of 109 (13%) were positive for Pular virus, indicating that this group of viruses may be able to switch host and infect humans more frequently than other bat-borne viruses, such as the Nipah virus [5]. Here, we describe the isolation and characterization of another new PRV strain, the Sikamat strain, which represents the seventh member in this species group (Table 1). This is the 4^th^ isolate from human patients within a four-year period in the absence of a targeted surveillance program. It can be predicted that more human cases will be detected if systematic surveillance is put in place for nations where fruit bats are present in significant numbers.

**Table 1:** List of known strains of *Pteropine orthoreovirus* (PRV).

Strain	Abbreviation	Isolation details			References
		Year	Host	Location	
Nelson Bay	PRV1NB	1970	Bat	Australia	[11]
Pulau	PRV2P	1999	Bat	Malaysia	[13]
Melaka	PRV3M	2006	Human	Malaysia	[5]
Kampar	PRV4K	2006	Human	Malaysia	[6]
HK23629/07	PRV5HK	2007	Human	Hong Kong	[7]
Xi River	PRV6XR	2010	Bat	China	[17]
Sikamat	PRV7S	2010	Human	Malaysia	This work

## Materials and Methods

### Collection of clinical samples as part of the influenza surveillance program

The investigation conducted in this study was approved by the ethics committee of the Malaysian National Public Health Laboratory. All patients (subjects) in this manuscript have given written informed consent (as outlined in the PLoS consent form) to publication of their case details. No identification of the subjects is to be revealed in any publication.

A nation-wide influenza surveillance program in Malaysia was initiated in 2004 under the national influenza pandemic preparedness plan. As a requirement, attending doctors from two respective designated government polyclinics per state were instructed to collect throat swab specimens from patients with influenza-like illnesses. Simultaneously, clinical and epidemiological information of the patients were recorded in a standardized questionnaire and sent together with the clinical specimens to the national public health laboratory for influenza virus isolation and identification.

### Virus isolation and preliminary identification

For virus isolation, throat swab samples in virus transport medium (VTM) were gently vortexed after the addition of crystalline penicillin (500 I.U.), streptomycin (200 µg) and amphoteracin (50 µg). After incubation for an hour at room temperature, the solution was inoculated in duplicates (100 µl and 200 µl, respectively) onto monolayer of MDCK (ATCC, CCL-34), Vero (ATCC, CCL-81) and Hep-2 (ATCC, CCL-23) cells freshly cultured in JM cell culture tubes [14]. The tubes were incubated at 37°C in an ambient of 5% CO_2_ and examined daily for evidence of any cytopathic effect (CPE). For those samples showing CPE, a 0.5 ml-aliquot of the culture supernatant containing suspended infected cells was carefully harvested and placed in a microfuge tube. The infected cells were pelleted and washed twice with sterile phosphate buffered saline (PBS) by centrifugation at 1000 *g* for 10 minutes. After the last wash, the infected cells were resuspended in 200 µl of sterile PBS and 10 µl of the suspension cells were carefully layered onto each well of a 12-well Teflon coated slide (Cel-Line/Erie Scientific Co., USA). The slide was allowed to dry over a warm plate (42°C) placed in a BSC-II cabinet. The slide was fixed for 10 minutes in cold acetone as soon as it was dried. Initial identification of virus was made by indirect immunofluorescence test using a panel of commercial monoclonal antibodies for identification of respiratory viruses commonly known to infect humans (Chemicon Inc., USA).

### Serological study

Virus infected cells fixed in wells of Teflon coated slides were used for serological investigation. Briefly, a fully confluent monolayer of MDCK cells in a 25-cm^2^ tissue culture flask was washed twice with 5 ml of sterile PBS and treated with 2 ml of 0.25% trypsin containing 0.1% EDTA (HyClone Laboratories Inc., Utah, USA). The MDCK cells were then suspended in 20 ml of DMEM culture medium containing 10% fetal calf serum. A 5-ml aliquot of the cell suspension was transferred into each of 4 new 25-cm^2^ flasks and incubated at 37°C in an ambient of 5% CO_2_. Once the cells reached confluent growth, the flasks were treated as above. The suspended cells in each flask were inoculated with 10^7^ TCID_50_ of each of the four viruses included in this study. At 24 hours post-inoculation, the infected cells (with full CPE) in the suspension were harvested and washed 5 times with sterile PBS by centrifugation at 1000 *g* for 10 minutes in each wash. A 25-cm^2^ flask of suspended mock-infected MDCK cells was processed exactly the same way as a negative control. After the last wash, the mock-infected cells were distributed and added into the respective infected cells in the ratio of 1∶4. The volume of the mixed cell suspension was adjusted with sterile PBS to a cell concentration of approximately 1000 cells per 10 µl and subsequently 10 µl of the final cell suspension was transferred into each well of the Teflon coated slides. The slides were allowed to air dry in a BSL-2 cabinet, irradiated by UV-light and fixed in cold acetone for later use.

Human sera were tested for the presence of virus-specific IgM and IgG antibodies by indirect immunofluorecence assay using the fixed infected cells prepared above. Convalescent human serum samples were serially diluted from 1∶20 to 1∶2560 with sterile PBS. Twenty microlitres of the diluted sera was used for immuno staining as previously described [15]. The end point titre of positive assay was taken as the highest serum dilution that gave at least five cells with positive immunofluorescence.

Cross neutralization activity of convalescent human sera was tested using a micro-neutralization test against 100 TCID_50_ of each of the four viruses using a previously published method [5].

### Molecular and sequence analysis

Extraction and purification of dsRNA, and genome segment analysis were carried our as previously described by our group [5], [6]. The majority of the sequence information was obtained by PCR with primers designed from conserved regions of known viruses of the PRV species group. To obtain the sequence of genome terminal ends, a seminested PCR was employed using a combination of genome-specific primers and the primer complementary to the adaptor ligated to the genome ends [6]. All regions of the genome segments were sequenced at least three times. Phylogenetic analysis was conducted using MEGA4 [16]. Neighbour-Joining trees were constructed with bootstrap values determined by 1000 replicates. Complete genome sequences of the four S- class genome segments were deposited in GenBank under accession numbers JF811580-3.

## Results

### Clinical presentations of the patient

A 46-year old policeman developed a sudden onset of high fever with a sore throat and prostrating myalgia on 6 March 2010. The fever was associated with severe anteriorly throbbing headache and mild photophobia, but without blurring of vision. There was no neck stiffness, abnormal muscle movement, seizure or loss of consciousness. The myalgia was described as generalized and associated with generalized body weakness, lethargy, malaise and loss of appetite. His sore-throat was described as severe in nature which was aggravated by swallowing of both solid and liquid food. The sore-throat was associated with hoarseness of voice and nasal blockage. There was no associated nasal discharge, cough or breathing difficulty. On the following day, he developed moderate abdominal pain over the epigastrium, but not associated with vomiting or diarrhoea. There was no history of skin rashes or a tendency to bleed. His illness was not relieved with self-medication of anti-pyretics.

He received medical treatment at a government health clinic on 8 March 2010. At the outpatient clinic, he was noted to be febrile (an axillary temperature of 39.8°C), ill-looking with mild erythematous flushing over his face and chest that blanched on pressure. He was noted to have mild conjunctivitis, but there was no jaundice or petechiae spots noted. His tonsils were enlarged and injected, but there was no white exudates noted over the oral pharynx. He was not in any respiratory distress and his lungs were clear with good bilateral air-entry on auscultation. Other systemic examinations were essentially normal and there was no significant enlarged lymph node noted. A provisional diagnosis of influenza-like illness was made. A throat swab was collected in viral transport medium and sent to the laboratory for virology investigation. He was given erythromycin and a higher dose of anti-pyretic. On follow-up on 12 March 2010, he still felt weak and lethargic with mild myalgia and poor appetite though his fever had resolved on 11 March 2010.

The patient had no past significant severe illness that needed hospitalization, neither was he on regular medication for any chronic illness. There was no history of recent travel overseas or into caves or jungles. There was no history of direct contact or handling of any domestic or wild animal or contact with neighbours or friends having similar illness. None of his other family members, living in the same household, developed similar illness within the month following his illness.

### Epidemiological findings

The patient and his family live in a double storey-linked house in Sikamat, a small town within the state of Negeri Sembilan, in the central western region of peninsular Malaysia. There is no fruit tree in the immediate surroundings of the house. However, for the past two years, the patient and his wife have regularly returned to stay over the weekend in their village house and were at the house on the weekends before the onset of his disease. The village house is situated in a 2-hectare orchard about 20 kilometres away from his main residence and is surrounded with various types of tropical fruit trees. Fruit bats are known to fly around in his orchard at night and at times were known to fly into the house, but none were found roosting inside the house. He has not directly handled any bats or killed any of them. He and his wife did not consume any fruit partially eaten by animal or insect or those drop on the ground. The patient has 6 children, two sons and four daughters. His two older sons, aged 22 and 20, occasionally assist him in the orchard during the weekend, but did not stay overnight in the village house. Samples of venous blood were collected from his wife, one son (2^nd^ child) and two daughters (5^th^ and 6^th^ child) on 20 April 2010 and the status of their anti-PRV7S antibodies is shown in Table 2. The patient's wife and 2^nd^ son had IgM and IgG antibodies to PRV7S though none of them had ever experienced any febrile illness within a month of the onset of the patient's clinical symptoms.

**Table 2:** Titer of anti-PRV7S antibodies in the sera of the patient and his family members.

Subjects	Sampling date	Serum antibody titer (dilution)	
		IgM	IgG
Patient	2010-03-22	1 : 40	1 : 640
Patient's wife	2010-04-10	1 : 20	1 : 320
Patient's 2^nd^ son	2010-04-10	1 : 40	1 : 320
Patient's 5^th^ daughter	2010-04-10	<1 : 10	<1 : 10
Patient's 6^th^ daughter	2010-04-10	<1 : 10	<1 : 10

### Laboratory findings

The throat swab sample from the patient in viral transport medium was processed for virus isolation as described in the method section. After 3 days of incubation, syncytium-forming CPE was noted in MDCK, but not in Hep-2 or Vero cells. After 2 passages in MDCK cells, the virus was able to replicate and cause similar CPE in all types of mammalian cell lines available in the laboratory (see SI Table 2 in reference [5]), including C6/36 (ATCC CRL-1660), a cell line of mosquito cell origin.

**Figure 1:**
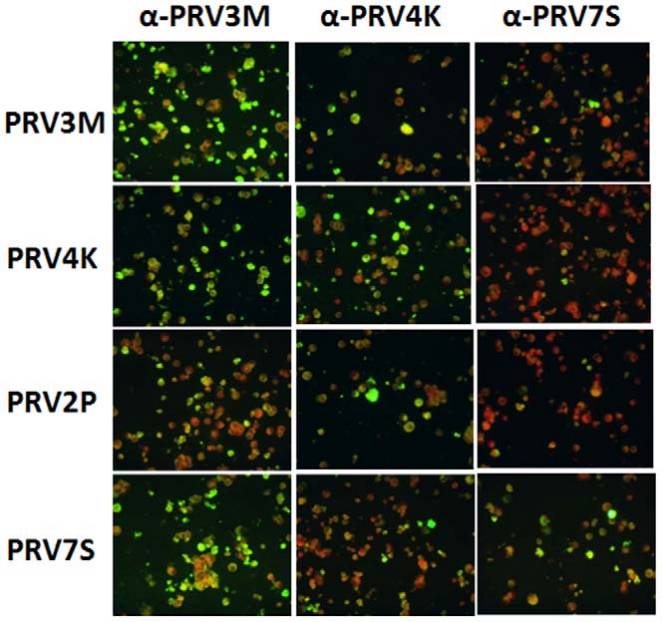
Immunofluorescence staining of four PRVs using human patient sera. MDCK cells infected with PRV3M, PRV4K, PRV2P and PRV7S (presented in panels from top to bottom), respectively, were each probed with convalescent serum from patient infected with PRV3M, PRV4K and PRV7S (from left to right), respectively. All human sera were used at 1∶40 dilution.

The infected MDCK cells failed to react with a commercial monoclonal antibody panel used for identification of commonly known respiratory viruses. On the other hand, the infected cells reacted strongly with sera derived from patients infected with PRV3M and PRV4K, respectively (data not shown). Further cross-reactivity analyses indicated that convalescent sera from patients infected with three different PRV strains all had high level of cross reactivity (Figure 1, Table 3). This was further confirmed by micro-neutralization assay (Table 3). It is worth noting that sera from patients infected with each of the three different PRVs also reacted with PRV2P, the only bat isolate of the PRV species group obtained in Malaysia so far.

**Table 3:** Antibody titer in patients' convalescent serum samples against each of the four PRV strains isolated in Malaysia.

Strain	Antibody titer in human convalescent seraIgG titer/neutralizing titer*		
	α-PRV3M(day 32)#	α-PRV4K(day 32)	α-PRV7S(day 16)
PRV2P	1∶320/1∶160	1∶320/1∶40	1∶160/1∶80
PRV3M	**1∶2560/1∶1280**	1∶640/1∶60	1∶320/1∶160
PRV4K	1∶640/1∶160	**1∶1280/1∶640**	1∶320/1∶160
PRV7S	1∶640/1∶320	1∶640/1∶160	**1∶640/1∶320**

*IgG titer was determined by immunofluorescence antibody test using fixed MDCK cells infected with each of the four viruses; neutralizing titer was determined using the micro-neutralization test with 100 TCID_50_ for each of the four viruses.

# Sampling time, expressed as days after onset of symptoms, is indicated for each serum.

### Molecular and phylogenetic characterization

**Figure 2:**
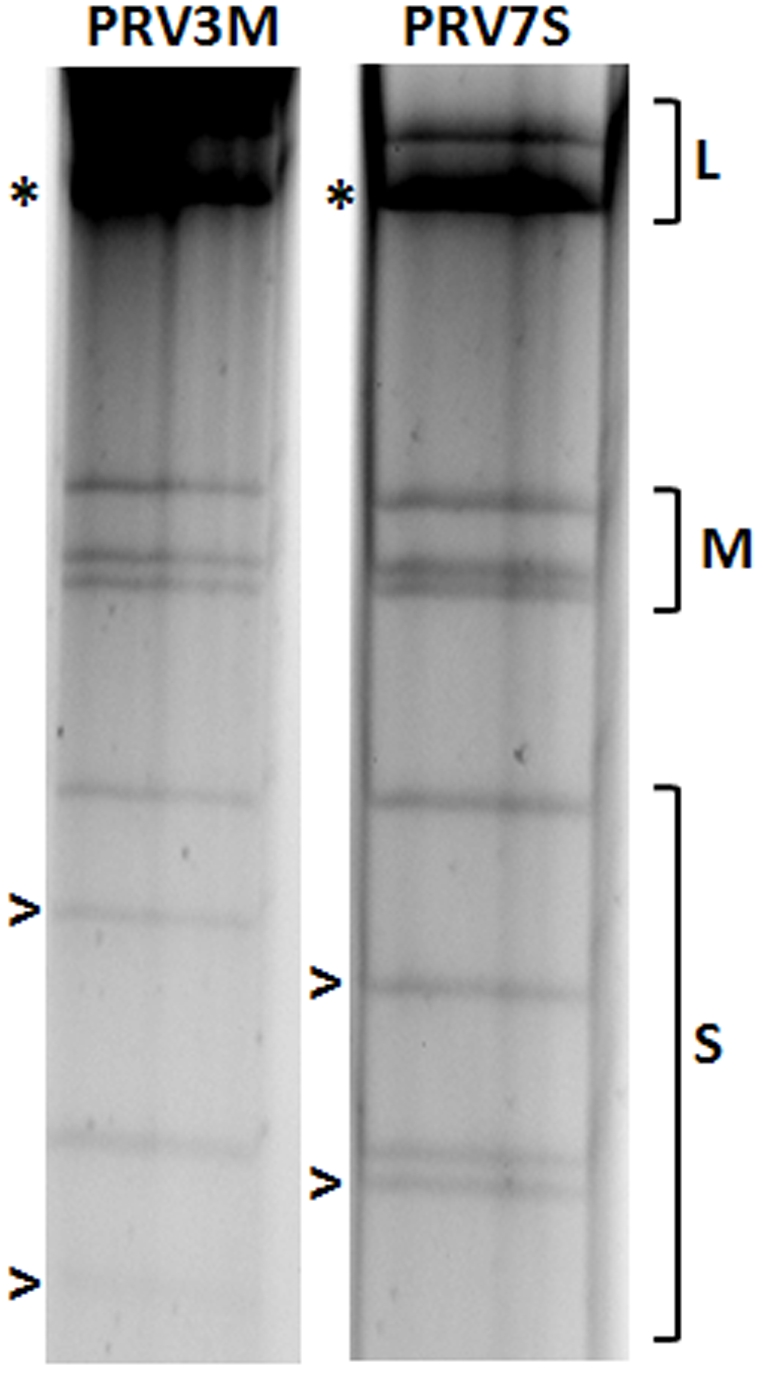
Comparison of genome segment profiles between PRV3M and PRV7S. The genome segments were separated in a 10% SDS-polyarylamide gel. The classes of genome segments (L, M and S) are labeled on the right. The asterisk (*) indicates co-migrating bands and the open arrow (>) indicates cognate segments of the two viruses with a different mobility.

Profiling of the genome segments for PRV7S on SDS-polyacrylamide gel indicated that its electropherotype is similar to those of other Malaysian PRVs, and is most closely related to that of PRV3M (Figure 2). Analysis of both nucleotide and deduced amino acid sequences for the four S- class segments of PRV7S showed high sequence relatedness to all known members of the PRV species group (Table 4). PRV7S can be confidently classified as a member of this species group based on the following molecular characteristics: (i) The deduced protein products encoded by the S- class genome segments of PRV7S are similar in size and share high level of sequence identity with all known members of the PRV species group (Table 4), including PRV6XR that was recently isolated from Rousettus bats in China [17]; (ii) The S1 genome segment of PRV7S is polycistronic and has identical coding arrangement for p10, p17 and σC proteins as seen in all members of the PRV species group, but different from other orthoreoviruses; (iii) The 5′ terminal consensus sequence is GCUUUA, which is identical for PRV species group members, but different from the other members of the genus *Orthoreovirus*.

**Table 4:** Comparison of sequence identity and size (aa residues) of PRV7S S-encoded proteins to those of PRV6XR, PRV5HK, PRV4K, PRV3M, PRV2P and PRV1NB.

PRV7S segment/protein (size in aa residues)	Percentage sequence identity to cognate PRV7S protein (size in aa residues)					
	PRV6XR	PRV5HK	PRV4K	PRV3M	PRV2P	PRV1NB
S1/p10 (95)	71% (95)	91% (89)	94% (95)	99% (95)	98% (95)	70% (95)
S1/p17 (142)	56% (147)	87% (142)	85% (142)	98% (142)	92% (142)	52% (140)
S1/σC (332)	48% (323)	66% (324)	56% (331)	97% (328)	80% (327)	42% (323)
S2/σ1 (416)	NA*	93% (391)	99% (416)	99% (416)	98% (416)	97% (416)
S3/σNS (367)	99% (367)	94% (347)	99% (367)	99% (367)	99% (367)	98% (367)
S4/σ2 (416)	NA*	97% (357)	97% (361)	99% (361)	94% (361)	94% (361)

*NA: sequence not available.

**Figure 3:**
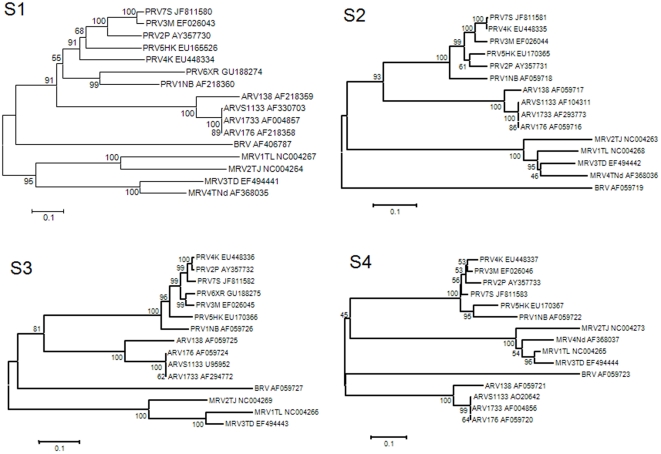
Phylogenetic trees based on the nucleotide sequence of the four S-class genome segments of orthoreoviruses. Abbreviations: ARV, Avian orthoreovirus; BRV, baboon orthoreovirus; DRV, Muscovy duck reovirus; MRV; Mammalian orthoreovirus. See Table 1 for the abbreviations of all seven Pteropine orthoreovirus strains known to date. The percentage of replicate trees in the bootstrap test (1000 replicates) is shown next to the branching node.

Phylogenetic trees based on all four S-class genome segments further confirmed that PRT7S is a member of the PRV species group (Figure 3).

## Discussion

The isolation of yet another orthoreovirus from a patient suffering acute respiratory disease in Malaysia confirms the trend of regular human infection by members of the *Pteropine orthoreovirus* (PRV) species group. Considering that the detection of such events was “accidental” in the sense that no targeted surveillance was conducted, it can be predicted that the real frequency of spillover will be higher.

To date, there are four confirmed outbreaks of human respiratory illness caused by this group of viruses, three in Malaysia and one in Bali/Hong Kong [5], [6], [7]. Although these viruses are genetically and serologically related, it is evident from the data presented in this and previous studies that each outbreak was caused by a different strain or isolate, some more closely related than others. Although there is a lack of direct evidence to prove a bat-to-human transmission, epidemiological studies indicated that patients from all four independent outbreaks had been to an area close to bat colonies or frequently visited by bats [5], [6], [7]. The most direct evidence came from the human infection by PRV3M, in which the patients' house was visited by a bat approximately a week prior to the onset of clinical disease in the index patient, followed by 3 additional human infections in the same family [5].

While there is the possibility of PRV infection in other wildlife animal(s), it is clear that bats are at least one of the natural reservoir hosts based on the fact that closely related PRVs have been isolated from three different bat species in three well separated geographic locations in Australia, Malaysia and China [11], [13], [17]. Based on phylogenetic analysis carried out for all of the PRV members known to date, it can also be concluded that there are a number of diverse viruses circulating among different bat populations, and similar viruses are capable of infecting bats from a different genetic background. As shown in Figure 3, among the three strains isolated from bats, PRV1NB (isolated from *Pteropus poliocephalus* in Australia), PRV2P (isolated from *Pteropus hypomelanus* in Malaysia) and PRV6XR (isolated from *Rousettus leschenaultia* in China), PRV1NB was clustered closer to PRV6XR than to PRV2P despite the fact that PRV1NB and PRV2P were isolated from bats in the same genus, *Pteropus*, while PRV6XR was from the genus *Rousettus*. The viral genetic diversity is also reflected by the four viruses isolated from human patients. Although they are closely related, they represented four independent spillover events associated with four distinctive viruses.

It is important to emphasize that among all three outbreaks investigated in Malaysia so far, each was associated with multiple human infections. Although one cannot exclude the possibility that this could be a result of independent bat-to-human transmission, epidemiological tracing suggested they were more likely the result of human-to-human transmission [5], [6]. This is also true for the cases presented in this study. In this context, it is important to note that among the multiple infections during each outbreak, there was at least one asymptomatic human infection [5], [6]. It is therefore possible that the first human case detected (the index case) may not be the first case of infection following spillover from a reservoir host.

In summary, recent studies demonstrated that there are a large number of genetically diverse PRVs circulating among different bats in geographically separated locations. Many, if not all of them, have the potential to infect human and cause respiratory disease outbreaks. These viruses can be transmitted between humans and the resulting clinical presentations vary with different individuals, from symptomless to severe respiratory illness or diarrhoea [5], [6], [7]. To date, there is no evidence of virus persistence among infected human populations. However, considering the great virus diversity in bats and asymptomatic infection in humans, it is possible for this group of viruses to adapt to become a common respiratory pathogen of humans.
